# Atypical Rho GTPases of the RhoBTB Subfamily: Roles in Vesicle Trafficking and Tumorigenesis

**DOI:** 10.3390/cells5020028

**Published:** 2016-06-14

**Authors:** Wei Ji, Francisco Rivero

**Affiliations:** 1 School of Life Science and Technology, Changchun University of Science and Technology, Changchun 130022, China; jiweicust@163.com; 2 Centre for Cardiovascular and Metabolic Research, Hull York Medical School, University of Hull, Cottingham Road, Hull, East Riding of Yorkshire HU6 7RX, UK

**Keywords:** cullin, cyclin E, HIFα, Rab9, RhoBTB, tumor suppressor, ubiquitination

## Abstract

RhoBTB proteins constitute a subfamily of atypical Rho GTPases represented in mammals by RhoBTB1, RhoBTB2, and RhoBTB3. Their characteristic feature is a carboxyl terminal extension that harbors two BTB domains capable of assembling cullin 3-dependent ubiquitin ligase complexes. The expression of all three *RHOBTB* genes has been found reduced or abolished in a variety of tumors. They are considered tumor suppressor genes and recent studies have strengthened their implication in tumorigenesis through regulation of the cell cycle and apoptosis. RhoBTB3 is also involved in retrograde transport from endosomes to the Golgi apparatus. One aspect that makes RhoBTB proteins atypical among the Rho GTPases is their proposed mechanism of activation. No specific guanine nucleotide exchange factors or GTPase activating proteins are known. Instead, RhoBTB might be activated through interaction with other proteins that relieve their auto-inhibited conformation and inactivated through auto-ubiquitination and destruction in the proteasome. In this review we discuss our current knowledge on the molecular mechanisms of action of RhoBTB proteins and the implications for tumorigenesis and other pathologic conditions.

## 1. Introduction

Ras superfamily small guanosine triphosphatases (GTPases) are important molecular switches that regulate a myriad of signaling pathways in eukaryotes. They characteristically cycle between an active GTP-bound state and an inactive GDP-bound state. Activation enables the GTPase to interact with a multitude of effectors that relay upstream signals to other cellular components. The members of the Ras superfamily can be distributed into several families, mainly Ras, Rho, Rab, Arf, Ran, and Miro, based on sequence similarities, each family regulating a major cellular process [1]. Within the Ras superfamily Rho GTPases are key regulators of the actin filament system and, consequently, of all processes that depend on the reorganization of the actin cytoskeleton, such as membrane trafficking, cell motility, cytokinesis, adhesion, and morphogenesis. They have also been found implicated in numerous processes not directly linked to actin reorganization, like NADPH oxidase activation, microtubule organization, gene expression, cell cycle progression, apoptosis, and tumorigenesis [2,3]. The GTPase cycle of Rho proteins is characteristically modulated by three major classes of proteins that are targets of upstream signaling pathways: guanine nucleotide exchange factors (GEFs) catalyze the exchange of GDP for GTP to activate the switch; GTPase activating proteins (GAPs) stimulate the (typically low) intrinsic GTPase activity and, thus, inactivate the switch; guanine nucleotide-dissociation inhibitors (GDIs) play complex roles stabilizing the GTPase and regulating exchange between membranes and cytosol.

Rho GTPases are ubiquitously expressed across the eukaryotes, frequently as multiple paralogs. In humans, the family comprises 21 members that have been grouped into subfamilies: Cdc42-like (Cdc42, TC10/RhoQ, TCL/RhoJ, Chp/Wrch-2/RhoV, Wrch-1/RhoU), Rac-like (Rac1–3, RhoG), Rho-like (RhoA–C), Rnd (Rnd1–2, Rnd3/RhoE), RhoD (RhoD and Rif/RhoF), RhoH/TTF, and RhoBTB (RhoBTB1–3) [4]. The RhoBTB subfamily stands out for three reasons that make these proteins truly atypical Rho GTPases. First, RhoBTB proteins have a peculiar domain architecture with a long extension following the GTPase domain. Second, their cellular roles bear no apparent relationship to direct remodeling of the cytoskeleton. Third, regulation of their activity may not involve specific GEFs and GAPs but interaction with other proteins that relieve their auto-inhibited conformation followed by auto-ubiquitination and destruction in the proteasome instead. The RhoBTB subfamily was identified during the study of the genes encoding Rho-related proteins in the lower eukaryote *Dictyostelium discoideum* [5]. Interest in RhoBTB arose when *RHOBTB2* (also called *DBC2*, deleted in breast cancer 2), was identified as the gene homozygously deleted in breast cancer samples and was proposed as a candidate tumor suppressor gene [6]. Subsequent studies have strengthened the implication of RhoBTB proteins in tumorigenesis and other cellular processes. In this review we shall present and discuss our current knowledge about the emerging functions of human RhoBTB proteins and their associated molecular mechanisms, with the occasional reference to studies carried out in mouse models.

## 2. RhoBTB Architecture and Gene Expression

**Figure 1:**
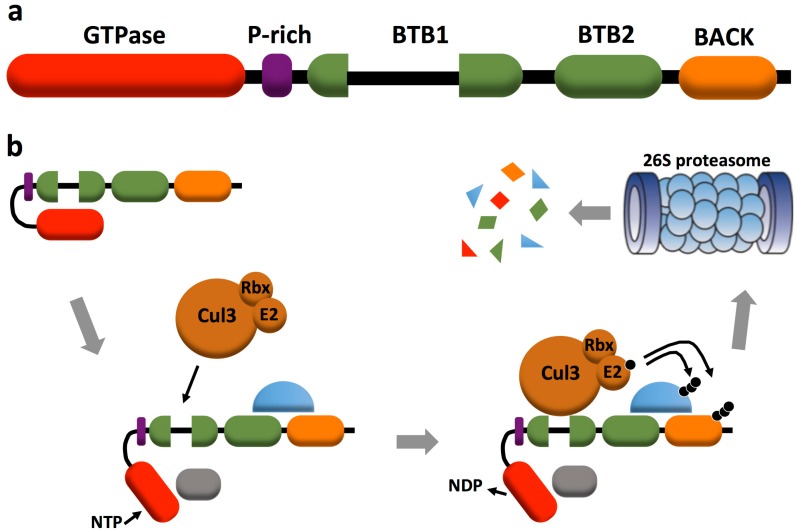
Architecture of RhoBTB proteins and proposed mechanism of regulation. (**a**) Domain structure of RhoBTB proteins. The GTPase domain is followed by a proline-rich region, a tandem of two BTB (broad complex, tramtrack, and bric-a-brac) domains (the first one is bipartite) and a carboxyl terminal BACK (BTB and C-terminal Kelch) domain. The cartoon is drawn roughly to scale and fits RhoBTB1 and RhoBTB2. RhoBTB3 has a shorter insert in the first BTB domain and it bears an isoprenylation (CAAX) motif at the end; and (**b**) a model depicting the hypothetical mechanism of activation and inactivation of RhoBTB. An intramolecular interaction of the GTPase domain with the first BTB domain maintains the molecule inactive. Interaction with specific ligands would provoke a conformational change that disrupts the intramolecular interaction. There is probably not a unique ligand binding site that causes activation of RhoBTB (two have been depicted in this model as examples). The GTPase domain would then be able to bind and hydrolyze GTP (in the case of RhoBTB2 and probably also RhoBTB1) or ATP (in the case of RhoBTB3) and the first BTB domain would be free to assemble a cullin 3-dependent ubiquitin ligase complex that would tag the ligands, as well as RhoBTB itself, for degradation in the proteasome. It has not been established whether nucleotide binding and cullin 3 binding are always linked.

The RhoBTB subfamily comprises three members: RhoBTB1 (696 amino acids), RhoBTB2 (727 amino acids), and RhoBTB3 (611 amino acids). RhoBTB1 and RhoBTB2 are very similar to each other (79%), whereas RhoBTB3 is the most divergent member (42% similarity to RhoBTB1 and RhoBTB2). RhoBTB proteins stand out among all other small GTPases for their domain architecture: the GTPase domain is followed by a proline-rich region, a tandem of two BTB (broad complex, tramtrack, and bric-a-brac) domains and a carboxyl terminal BACK (BTB and C-terminal Kelch) domain (Figure 1a). A detailed analysis of the domain architecture of RhoBTB has been discussed in a previous review [7]. Here we shall present a summary of the key features that incorporates some novel aspects.

The GTPase domain of RhoBTB1 and RhoBTB2 is Rho-related, but contains some deviations from the GTPase consensus of most Rho GTPases. These include a longer than usual Rho insert region, two insertions placed one immediately before (six residues) and one after (10 residues) switch I, and a deletion (two residues) that affects the phosphate/magnesium binding region 3 within switch II. In addition the glycine residue equivalent to G12 in Ras appears substituted by asparagine. These deviations do not prevent binding of GTP, as has been shown recently for RhoBTB2, and RhoBTB1 is likely to behave in the same way [8]. By contrast, the GTPase domain of RhoBTB3 is considerably divergent and binds and hydrolyzes ATP instead of GTP by virtue of some critical amino acid replacements in the G4 and G5 motifs [9]. RhoBTB3 branches outside the Rho family when phylogenetic analysis takes into consideration the GTPase domain only. However analyses of a large sample of RhoBTB proteins from a wide spectrum of species provide compelling phylogenetic evidence for grouping this protein within the RhoBTB subfamily [10]. The proline-rich region links the GTPase to the first BTB domain and is a potential SH3 domain-binding site. BTB domains participate in homomeric and heteromeric associations with other BTB domains and function as components of multimeric cullin 3-dependent ubiquitin ligase complexes. The first BTB domain of RhoBTB is bipartite, carrying an insertion of unknown function. This domain binds to cullin 3 and to the GTPase domain. The BTB domains of RhoBTB allow the formation of homodimers and of heterodimers with other proteins of the RhoBTB subfamily [11]. The carboxyl terminal region exhibits similarity to a particular motif (the 3-box) of the BACK domain mainly found in BTB proteins with kelch domains and probably involved in substrate orientation in cullin 3-based ubiquitin ligase complexes [12]. Only RhoBTB3 bears an isoprenylation CAAX motif that is typical for classical Rho GTPases and contributes to, but is not the only determinant for the localization of the protein to membranes of the Golgi apparatus [11,13].

The expression patterns of all three RhoBTB encoding genes, both in human and mouse, have been discussed in a previous review [7]. Briefly, all three genes are ubiquitously expressed, although each with a specific organ pattern. Expression of *RHOBTB2* is generally weaker than the other two. All three genes are expressed in fetal tissues. Expression of the mouse *Rhobtb3* gene has been investigated in great detail at tissue rather than whole organ level in a gene trap mouse strain that expresses a β-galactosidase reporter under the control of the endogenous *Rhobtb3* promoter. Expression of this gene is nearly ubiquitous in the embryo, with particularly high levels in bone, cartilage, all types of muscle, testis, and restricted areas of the nervous system. In the adult mouse, expression declines considerably, but persists at low levels in cardiac muscle, the tunica media of blood vessels, the muscularis of hollow organs and cartilage, and at high levels in the seminiferous tubules and peripheral nerves [14]. Expression levels of *RHOBTB* genes change during the cell cycle, in tumors and in other circumstances that are described in following sections.

**Figure 2:**
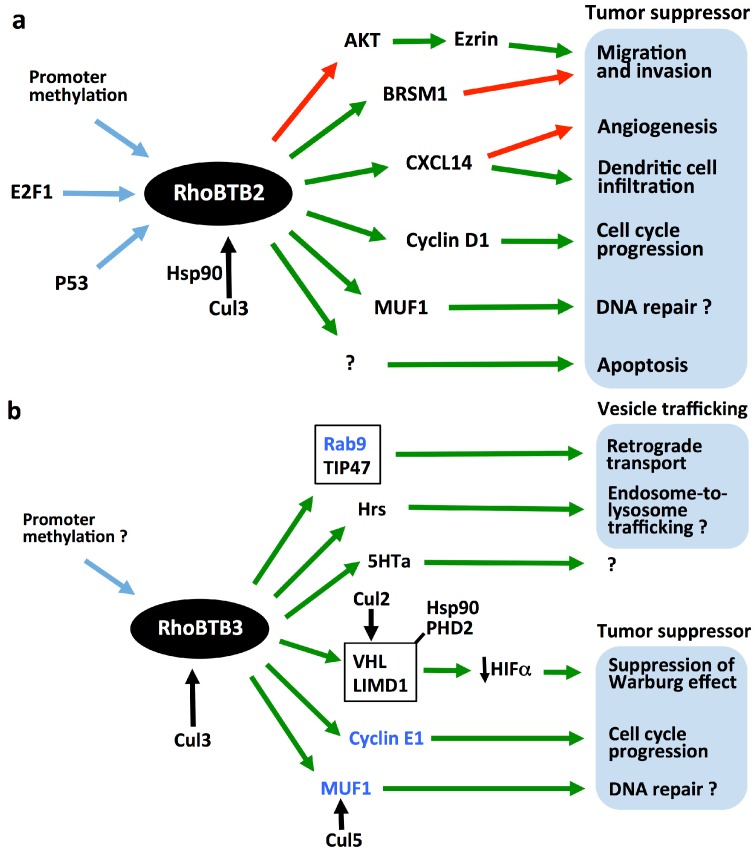
Schematic illustrating the roles of RhoBTB proteins in tumorigenesis and other processes. Proposed mechanisms for (**a**) RhoBTB2 and (**b**) RhoBTB3 are shown. RhoBTB1 (not depicted) is likely to function similarly to RhoBTB2 but has not been investigated extensively. Blue arrows indicate factors that affect the expression of the RhoBTB-encoding gene. Green arrows indicate positive or stimulatory links. Red arrows indicate inhibitory links. Interacting cullins are indicated with black arrows. The arrows do not implicate direct interactions; see Table 1 for details on interactions. Recognized substrates of RhoBTB3-dependent ubiquitin ligase complexes are shown in blue. Multiprotein complexes are boxed.

In what follows we shall discuss the roles of RhoBTB proteins in proteasomal degradation, tumorigenesis and vesicle trafficking, summarily presented in Figure 2, and shall introduce several interacting partners listed in Table 1.

## 3. RhoBTB and Proteasomal Degradation

The identification of the BTB domain as adaptor in cullin 3-dependent ubiquitin ligase complexes in 2003 prompted investigations into the participation of RhoBTB proteins in those complexes. We know now that all three RhoBTB proteins interact specifically with cullin 3 and that the interaction involves the first BTB domain and the amino terminal region of cullin 3 [11,15]. RhoBTB proteins may potentially interact with the amino terminal region of cullin 5, too, as shown by a yeast two-hybrid approach, but this interaction is apparently not favored *in vivo*. Cullin 3-dependent RhoBTB complexes seem to incorporate other cullins, as shown in immunoprecipitation and colocalization studies [11], reflecting the fact that cullins can heterodimerize and that some RhoBTB-interacting proteins recruit themselves other cullin complexes. Such is the case of MUF1 (LLRC41, leucine rich repeat containing 41) and VHL (von Hippel-Lindau protein) discussed below (Figure 2b). RhoBTB proteins not only target other proteins for ubiquitination, but they are, themselves, substrates for the cullin 3-based ubiquitin ligase complexes they form [11,15]. Berthold *et al.* proposed an autoregulatory model for RhoBTB proteins that has gained support in recent years [11]. According to this model, RhoBTB proteins exist in an inactive state through an intramolecular interaction of the first BTB domain with the GTPase domain. This intramolecular interaction prevents binding to cullin 3 and the formation of a ubiquitin ligase complex. It was proposed that the interaction with other proteins, including potential substrates, would relieve the autoinhibitory conformation of RhoBTB3 and facilitate the binding to cullin 3 (Figure 1b).

Several potential substrates or activators of RhoBTB have been described in the last years. MUF-1 is able to interact with all three RhoBTB proteins and is degraded in the proteasome by a RhoBTB3-cullin 3-dependent ubiquitin ligase complex [19]. Cyclin E, too, is targeted for ubiquitination by a RhoBTB3-cullin 3-dependent ubiquitin ligase complex [13]. RhoBTB3 also participates in the degradation of HIFα (hypoxia inducible factor α) through a multimolecular complex that involves VHL [18]. MUF1, cyclin E and HIFα have implications in tumorigenesis and are discussed below in more detail. RhoBTB3 also interacts with the 5-HT7a receptor, the most common splice variant of the serotonin receptor 7. This receptor is involved in a wide variety of pathophysiological processes of the central nervous system. Interestingly, the 5-HT7a receptor interacts with cullin 3 independently of RhoBTB3, and RhoBTB3 apparently inhibits proteasomal degradation of the receptor [17].

The autoinhibition model outlined above has received one more tweak in recent years. Manjarrez *et al.* identified RhoBTB2 as an interactor of the Hsp90 chaperone machinery in an immunoprecipitation screening and showed that this multimolecular complex unlocks RhoBTB2, enabling GTP binding [8]. The interaction of RhoBTB2 with cullin 3 is also dependent on Hsp90 activity, as it was significantly reduced in the presence of the Hsp90 inhibitor geldanamycin. Further affinity purification studies with a tagged RhoBTB2 yielded the majority of components of the COP9 signalosome, a multiprotein complex that binds to, and regulates, cullin 3 [20]. The Hsp90 chaperone machinery undergoes a complex ATP-driven reaction cycle that involves the binding and detachment of various components [21]. Using a biochemical approach with various inhibitors of Hsp90 Manjarrez *et al.* worked out a model in which RhoBTB2 associates to an intermediate Hsp90 complex that upon binding of ATP releases some components and breaks the intramolecular interaction of the GTPase domain of RhoBTB2 with the first BTB domain. This conformational change enables binding of GTP and cullin 3 to their respective domains. The Hsp90 chaperone is then released from the RhoBTB2-cullin 3 complex, allowing components of the COP9 signalosome to assemble and regulate the ubiquitin ligase complex [8]. Considering the high degree of similarity between RhoBTB2 and RhoBTB1, this mechanism is very likely to apply to RhoBTB1, too. Although Hsp90 does not seem to interact with RhoBTB3, it is incorporated in a RhoBTB3-dependent complex that targets HIFα through interaction with the prolyl hydroxylase PHD2 and may play a similar stabilizing role [18].

## 4. RhoBTB and Vesicle Trafficking

Although available antibodies fail to recognize any endogenous RhoBTB in fixed cells and tissues, studies with ectopically- expressed epitope-tagged proteins have revealed a vesicular pattern, frequently in the proximity of microtubules for RhoBTB2 [11,22] and predominantly surrounding the centrosome for RhoBTB3 [9,11]. Most RhoBTB3 co-localizes with Golgi apparatus markers but some co-localizes with early endosome markers or in close vicinity to microtubules or stress fibers [9,11]. These, and other early studies, suggested a participation of RhoBTB proteins in vesicle trafficking processes (reviewed in [7]). Compelling evidence for a role in these processes, more specifically in retrograde transport from endosomes to the Golgi apparatus, has been provided for RhoBTB3.

Espinosa *et al.* identified RhoBTB3 as an interacting partner of activated Rab9 in a yeast two-hybrid screen [9]. The interaction is specific for this Rab out of 54 Rabs tested. Rab9 is present on late endosomes and on transport vesicles that travel from late endosomes toward the Golgi complex. Mannose 6-phosphate receptors are recycled following this retrograde transport route, which requires Rab9, the cargo selection protein TIP47, cytoplasmic dynein, additional small GTPases, and a SNARE complex [23]. RhoBTB3 appears as a component of this retrograde transport complex and appears to interact also with TIP47 [9]. When RhoBTB3 is depleted by gene silencing, the Golgi apparatus becomes fragmented [13], the mannose 6-phosphate receptor adopts a disperse localization in Rab9 positive vesicles, and the secretion of the lysosomal enzyme hexosaminidase increases, but endocytosis and exocytosis are not changed, all indicative of a specific alteration of retrograde transport [9]. Based on these and additional experimental evidence, the authors proposed a model in which Rab9 on vesicles travelling from late endosomes to the Golgi apparatus relieves the autoinhibitory conformation of RhoBTB3 and allows maximal ATP hydrolysis. Activation of RhoBTB3 then releases TIP47, facilitating vesicle uncoating and membrane fusion.

RhoBTB3 also binds to the ubiquitin-interacting motif of Hrs (hepatocyte growth factor-regulated tyrosine kinase substrate), an early endosome protein that controls endosome-to-lysosome trafficking and may, therefore, participate in sorting of membrane cargo proteins to multivesicular bodies for subsequent degradation in the lysosome, an aspect of the RhoBTB3 physiology that requires further investigation [16].

## 5. RhoBTBs as Tumor Suppressors

Consistent with roles as tumor suppressors, expression of all three *RHOBTB* genes has been found reduced or extinguished in a variety of tumor types. Expression of *RHOBTB2*, the first member of the family to be proposed as a candidate tumor suppressor gene, has been found decreased in breast [6,24,25], lung [26], bladder [27,28], and stomach [29] cancers, in osteosarcomas [30], as well as in cell lines derived from breast, lung [6], and bladder [27] tumors, and HNSCCs (head and neck squamous cell carcinomas) [31]. Very often the degree of expression correlates with the primary location, grading, staging, or other features of the tumor. Expression of *RHOBTB1* has been found decreased in kidney, breast and stomach tumors in a cancer profiling array [11], as well as in HNSCCs [32] and in colon cancer tissues [33]. Expression of *RHOBTB3* has been found moderately but significantly decreased in breast, kidney, uterus, lung, and ovary tumor samples in a cancer profiling array [11] and in various subtypes of renal cell carcinomas [18]. Interestingly, the expression changes of *RHOBTB1* and *RHOBTB3* correlated with those of *CUL3* (the gene encoding cullin 3) in the same samples.

Decreased *RHOBTB* mRNA levels are rarely caused by mutations. The reported rare mutations and other genetic alterations have been described in detail in a previous review [7]. Epigenetic alterations, more specifically promoter methylation, is one potential mechanism to account for the reduced expression of these genes in tumors. The promoter regions of all three *RHOBTB* genes have a high GC content with CpG islands. The hypermethylation of CpG islands results in the downregulation or complete abrogation of gene expression and is a frequent epigenetic alteration in primary tumors [34]. *RHOBTB2* promoter methylation, normally a rare event, has been found increased and correlated to reduced or abolished expression of the gene in breast [25,35,36] and bladder [28] cancers. *RHOBTB2* promoter methylation appears to associate with more advanced tumor stages [25] or a particular status, like the presence of p53 mutation, HER2-positive status [25], or progesterone receptor negative status [36]. In fact, methylation of *RHOBTB2* and other genes in peripheral blood is a potential epigenetic marker for predicting the risk of breast cancer development [37].

A different mechanism of downregulation is provided by the action of microRNAs, a family of regulatory RNAs whose expression levels are altered in many types of cancer [38]. Two studies have identified *RHOBTB1* as a target of the microRNA miR-31. In one study overexpression of miR-31 in esophagus caused by zinc deficiency, a risk factor for the development of esophageal squamous cell carcinoma, was found associated to down-regulation of *RHOBTB1* [39]. In another study it was found that silencing of *RHOBTB1* in the colon cancer cell line HT29 mimics the effects of increased miR-31 expression, namely increase of cell proliferation and promotion of cell clonal growth, suggesting that downregulation of *RHOBTB1* is responsible for the tumor-promoting effects of miR-31 [33].

## 6. Tumorigenic Mechanisms of RhoBTB

### 6.1. Regulation of the Cell Cycle

Mounting evidence is beginning to shed light into the molecular mechanisms of the tumor suppressor activity of RhoBTB proteins. Although many aspects remain speculative, regulation of the cell cycle is an emerging common theme that is gaining wider support. The effects of RhoBTB2 on cell proliferation were observed in early studies where overexpression of the protein in the breast cancer cell line T-47D (a cell line that lacks *RHOBTB2* transcripts) effectively suppressed cell growth and increased the apoptotic ratio *in vitro* [6,40]. Similarly, overexpression of RhoBTB2 in osteosarcoma cells significantly arrested cells at G1 and resulted in apoptosis [30]. More puzzling is a recent report showing that in the thyroid carcinoma cell line SW579 treatment with recombinant RhoBTB2 for 24 h inhibited proliferation and provoked an increase of the apoptotic ratio through the mitochondrial apoptotic signaling pathway [41]. This report provides no explanation as to how the exogenously added RhoBTB2 exerts those actions. The growth arrest effect of RhoBTB2 on T-47D cells has been explained by the downregulation of cyclin D1. Cyclin D1 is upstream of cyclin E and the overexpression of any of both prevented the growth arrest effect of RhoBTB2 [42]. The effect on cyclin D1 is only partially dependent on proteasomal degradation [43]. It has not been investigated whether cyclin D1 or any other cyclin is targeted by RhoBTB2 for ubiquitination.

Further in support for roles of RhoBTB2 in cell cycle regulation, *RHOBTB2* has been identified as a target of the E2F1 transcription factor [44]. RhoBTB2 levels increase upon initiation of prophase and decrease at telophase, and this effect depends on E2F1. RhoBTB2 levels also increase during drug-induced apoptosis in an E2F1-dependent manner. Consequently, long-term overexpression of *RHOBTB2* had a negative effect on cell cycle progression and proliferation, and the downregulation of *RHOBTB2* delayed the onset of apoptosis. Of note, *RHOBTB2* has also been identified as a p53 candidate target gene, but the implications have not been investigated [45]. Although there are no studies addressing RhoBTB1 specifically, much of what we know about RhoBTB2 is likely to apply to RhoBTB1 because of their similarity.

A clear link between RhoBTB and the regulation of the cell cycle has been described in a recent study from the Pfeffer lab [13]. The observation that depletion of RhoBTB3 caused increased cell size and signs of genomic instability prompted these investigators to explore possible alterations of the cell cycle. It was observed that depletion of RhoBTB3 causes S-phase arrest in cultured cells accompanied by increased levels of cyclin E and increased activity of its dependent kinase CDK2. Cyclin E regulates the cell cycle transition from G1 to S phase and is degraded before entry into G2 phase by two independent cullin-mediated pathways, a cullin 1-dependent SCF-FBW7 pathway that targets phosphorylated cyclin E, and a less-well characterized cullin 3 pathway that targets free, unphosphorylated cyclin E [46]. RhoBTB3 binds cyclin E1 (and to a lesser extent cyclin B1) and this interaction is not coupled to binding of the cyclin to CDK2. RhoBTB3 then targets cyclin E for ubiquitination by a cullin 3-dependent ubiquitin ligase complex that resides at the Golgi apparatus. Similar to RhoBTB2, RhoBTB3 protein levels fluctuate along the cell cycle, with an accumulation during the S phase after the plateau of cyclin E [13]. Deregulation of cyclin E levels is common in tumor cells and can have a significant impact on cell proliferation, as shown in breast cancers where high cyclin E correlates consistently with poor prognosis [47].

### 6.2. Modulation of the Adaptive Response to Hypoxia

Zhang *et al.* have, very recently, described a novel function and potential tumorigenic mechanism for RhoBTB3. The authors of this study identified RhoBTB3 as an interacting partner for VHL in a yeast two-hybrid screen [18]. VHL is a tumor suppressor that normally functions as a component of a cullin 2-dependent ubiquitin ligase complex that targets hydroxylated HIF. HIFs are key regulators of adaptive responses to low oxygen concentration. In the presence of oxygen their α-subunits are rapidly degraded after hydroxylation by the prolyl oxidase PHD2. Under hypoxia conditions HIFs accumulate and bind to hypoxia responsive elements of various genes, in many cases related to aspects of cancer growth. In fact, aberrant accumulation or activation of HIFs is closely linked to many types of cancer [48]. RhoBTB3 acts as a scaffold for a multicomponent complex that contains PHD2 and VHL and facilitates ubiquitination of HIFα. Additionally RhoBTB3 appears to heterodimerize with LIMD1, an adaptor for PHD2 and VHL, and this interaction enhances the activity of the complex. The chaperone Hsp90 is incorporated to the complex through interaction with PHD2 and although it does not seem to interact directly with RhoBTB3, it may contribute to relieve its autoinhibitory conformation, as proposed for RhoBTB2 and discussed above [8]. Hypoxia apparently reduces the formation of the RhoBTB3-dependent multicomponent complex, resulting in an accumulation of HIFα.

Evidence that RhoBTB3 functions as a tumor suppressor has been provided in xenograft experiments with Ras-transformed embryonic fibroblasts isolated from Rhobtb3 deficient mice or HeLa cells in which *RHOBTB3* was silenced. The xenografts derived from RhoBTB3 deficient or silenced cells were larger and had increased levels of HIFα and their gene targets compared to those from control cells [18]. The authors proposed that RhoBTB3 inhibits tumorigenesis by maintaining low HIFα levels and consequently suppressing the Warburg effect, a shift towards high rate of glycolysis and lactic acid fermentation that characterizes most cancer cells.

### 6.3. Other Potential Tumorigenic Mechanisms

Further studies have provided pieces of evidence for additional potential tumorigenic mechanisms of RhoBTB proteins, but they are not as well investigated as the mechanisms described above for RhoBTB3. McKinnon *et al.* reported a decrease in the chemokine CXCL14 mRNA expression upon silencing of *RHOBTB2* in primary lung epithelial cells. The same effect was elicited by silencing *RHOBTB1* or *RHOBTB2* in keratinocytes, and conversely, expression of RhoBTB2 in HNSCCs restored CXCL14 expression. These effects are apparently independent of cullin 3-mediated protein degradation [31]. CXCL14 controls dendritic cell infiltration and angiogenesis and its expression is frequently lost in diverse epithelial tumors, including most HNSCCs [49].

Ling *et al.* reported that ectopic expression of *RHOBTB2* in two human metastatic breast cancer cell lines, MDA-MB-231 and MDA-MB-435, inhibits cell migration and invasiveness through a mechanism that involves upregulation of BRMS1 (breast cancer metastasis suppressor 1) and decreased phosphorylation of ezrin and Akt2 [50]. Ezrin is a cytoskeleton and signaling molecule that regulates cell adhesion, migration, and invasion, whereas Akt2 is a kinase involved in invasiveness of breast cancer cells and is able to phosphorylate ezrin [51].

Finally, Schenková *et al*, identified MUF1 in a two-hybrid screening for RhoBTB3 binding partners [19]. MUF1 is a ubiquitously expressed nuclear protein and carries a BC-box that functions as a linker in multicomponent cullin 5-dependent ubiquitin ligase complexes, followed by a LLR region involved in dimerization. MUF1 co-immunoprecipitates all three RhoBTB proteins and may be a substrate for RhoBTB-cullin 3 ubiquitin ligase complexes independently of its interaction with cullin 5. The function of MUF1 is unknown, but it becomes phosphorylated by ATM (ataxia telangiectasia mutated)/ATR (ATM and Rad3 related) upon DNA damage and is suspected to be involved in the DNA damage response [19]. Thus, MUF1 provides an additional tumorigenic mechanism for RhoBTB proteins worth exploring and together with the RhoBTB3-VHL example discussed above, one more example of crosstalk between cullin-dependent ubiquitination pathways.

## 7. Implications in Other Diseases and Animal Models of RhoBTB Function

While evidence for participation of RhoBTB proteins in vesicle trafficking and tumorigenesis is mounting, and the associated molecular mechanisms are being elucidated, additional roles are emerging that still require further investigations. Consistent with the pattern of expression of the gene encoding RhoBTB3 in selected areas of the nervous system, roles for this protein in neurological processes can be anticipated. Large scale transcriptional studies suggest that RhoBTB3 might be implicated in the development of psychotic disorders and Alzheimer’s disease. Using a convergent functional genomics approach Kurian *et al.* identified *RHOBTB3* as a candidate blood biomarker for psychotic disorders. *RHOBTB3* gene expression was found decreased in the blood of patients with high hallucination states [52]. A previous gene expression profiling study found decreased *RHOBTB3* expression in the frontal cortex of schizophrenic patients who completed suicide [53]. *RHOBTB3* has also been proposed as a candidate vulnerability gene for Alzheimer’s disease, vulnerability being defined as higher expression in the CA1 *versus* CA3 region of the hippocampus and increased expression in disease [54].

RhoBTB1 has emerged recently as a component of a signaling mechanism that regulates vascular function and blood pressure. *RHOBTB1* has been identified as a target gene of the nuclear hormone receptor PPARγ (peroxisome proliferator-activated receptor γ) and binding sites for this receptor have been found in the *RHOBTB1* locus [55]. PPARγ is a well-known regulator of adipogenesis, but also has antihypertensive and vascular protecting effects directly in the vascular endothelium and smooth muscle [56]. RhoBTB1 mRNA and protein levels are decreased in the aorta of mice expressing a dominant negative PPARγ. These mice also present a concomitant decrease in cullin 3, and it has been proposed that RhoBTB1 regulates cullin 3 levels or activity which, in turn, regulates RhoA turnover in smooth muscle [55]. RhoA turnover is mediated by a cullin 3-dependent complex, but the specific role of RhoBTB1 in this complex remains to be elucidated.

A single case of a male carrying a balanced paracentric inversion of chromosome 5 that disrupts *RHOBTB3* has been published. This patient presented asymmetric leg growth and large hands, and behavior problems. It has not been determined whether disruption of *RHOBTB3* is the cause of those alterations [57]. As mentioned above, a few pathogenic mutations of *RHOBTB2* have been described but they affect only tumors. No mutations have been reported for *RHOBTB1*.

Almost all the information about the roles of RhoBTB proteins discussed until now has been gained from studies *in vitro* or in cells in culture. To understand the function of these proteins at the whole organism level one needs to resort to animal models. To date only a mouse model of *Rhobtb3* disruption (a gene trap knockout) has been characterized to some extent. Disruption of the *Rhobtb3* gene causes reduced perinatal viability, a postnatal growth defect that persists in males after weaning and reduced testis size [14]. The reduced testis size is likely associated to the marked fertility defect that characterizes *Rhobtb3* deficient males, but females are also present reduced fertility [58]. Standard tests performed by the Sanger Centre, where the mouse model was created, revealed reduced grip strength, suggesting roles in neural processes and consistent with localized expression of *Rhobtb3* in areas of the central nervous system and in peripheral nerves. Platelets isolated from *Rhobtb3*-deficient mice present aggregation and degranulation defects, but their morphology is unaffected. These defects may be caused by subtle alterations of the granulogenesis process in the megakaryocyte, which is dependent on correct functioning of the Golgi apparatus [59].

Lack of RhoBTB3 did not affect the rate of proliferation of primary lung fibroblasts isolated from 10-week-old animals [14], but higher proliferation rates have been reported in mouse embryonic fibroblasts [18]. Ablation of *Rhobtb3* only caused very modest changes in the pattern of gene expression of adult heart and brain [14]. Collectively these observations seem to indicate that RhoBTB3 plays more prominent roles at early stages of development, consistent with the pattern of expression of the Rhobtb3 gene in the mouse.

## 8. Conclusions

The molecular mechanisms of action of RhoBTB proteins are beginning to be elucidated and, in most cases, revolve around their role in ubiquitination and proteasomal degradation. As it appears, RhoBTB proteins cannot be understood outside multiprotein complexes, several examples of which have been uncovered in recent years. The evidence gathered to date also supports the autoregulatory model proposed in early studies while suggesting that there is not a unique pattern of interaction and activation of RhoBTB (Figure 1b). For example, MUF1 binds the carboxyl terminal region of RhoBTB3 downstream of the GTPase domain [19], whereas cyclin E requires residues at the amino and carboxyl terminus [13] and Rab9 binds to the carboxyl terminal region downstream of the first BTB domain [9]. The interaction of RhoBTB2 with components of the Hsp90 chaperone is complex and requires the GTPase domain and motifs downstream of this domain [8].

Some of the tumorigenic mechanisms of RhoBTB proteins are starting to take shape, in particular the participation in the regulation of the cell cycle. RhoBTB3 appears to have a dual function at the Golgi apparatus, regulating vesicle trafficking and the S/G2 transition of the cell cycle through different sets of interactions [9,13]. It remains to be established, at least for RhoBTB3, whether some of their functions are independent of the formation of cullin 3-dependent ubiquitin ligase complexes. Notably, the studies outlined in this review have revealed several instances of crosstalk between complexes dependent on various cullins (Figure 2b).

Apart from their unusual domain architecture and their roles, another aspect makes RhoBTB proteins atypical, namely their possible mechanisms of activation and inactivation. There are no known GEFs or GAPs for these proteins, but the current evidence suggests that activation requires the interaction with particular ligands. Rab9 and Hsp90 bind to the respective RhoBTB and facilitate nucleotide binding and in the case of Hsp90 assembly of a cullin 3-dependent complex and could, therefore, be considered activators. Other proteins, like MUF1 and cyclin E are possibly substrates that bind to previously activated RhoBTB-cullin 3 complexes. A likely mechanism for inactivation of RhoBTB proteins would involve auto-ubiquitination by the cullin 3-dependent ubiquitin ligase complexes they form, followed by proteasomal degradation (Figure 1b). This is not an unusual mechanism, as other Rho GTPases are to some extent modulated by proteasomal degradation [60].

Although considerable progress has been made in recent years towards deciphering the mechanisms of action and regulation of RhoBTB proteins, future work will hopefully help to achieve a deeper understanding of the functioning of these proteins and their participation in the pathogenesis of cancer and other conditions.

## Figures and Tables

**Table 1:** Interaction partners of RhoBTB and their functions. The technique used to identify the interaction is given in brackets. B2H, bacterial two hybrid; CL, chemical crosslinking; IP: immunoprecipitation; IVEC, *in vitro* expression cloning followed by GST pull-down; Y2H, yeast two-hybrid. Note that some interactions included in the table, particularly those found using only immunoprecipitation, are not necessarily direct interactions. The RhoBTB3/LIMD1-PHD2-VHL-HIFα complex and the RhoBTB2/Hsp90 complex have not been investigated in detail in terms of direct interactions. Hsp90 is part of the above RhoBTB3 complex, but through PHD2, not directly. Hsp90 chaperone refers to Hsp90 and co-chaperone components. Note also that RhoBTB proteins are capable of homo and heterodimerization.

Protein	RhoBTB	Function	Reference
Cullin 3	1, 2, 3 (Y2H, IP)	E3 ubiquitin ligase component.	[8,11,15]
Cullin 5	2, 3 (Y2H)	E3 ubiquitin ligase component.	[11]
Cyclin E1, cyclin B1	3 (IP)	Regulation of the cell cycle.	[13]
Hrs	3 (IVEC)	Protein sorting for lysosomal degradation.	[16]
5-HT7a	3 (Y2H)	Serotonin receptor.	[17]
Hsp90 chaperone	2 (IP); 3 (IP)	Protein folding and stabilization.	[8,18]
LIMD1	3 (IP)	Multifunctional scaffold protein.	[18]
MUF1	1, 2, (IP); 3 (B2H)	Adaptor for cullin 5-dependent ubiquitin ligase complexes.	[19]
PHD2	3 (IP)	Prolyl hydroxylase.	[18]
Rab9A, Rab9B	3 (Y2H)	Retrograde transport of membrane receptors.	[9]
TIP47	3 (CL)	Cargo packaging for endosomal transport.	[9]
VHL	3 (Y2H, IP)	Adaptor for cullin 2-dependent ubiquitin ligase complexes.	[18]
